# Surgical outcomes with high and low pulse energy femtosecond laser systems for cataract surgery

**DOI:** 10.1038/s41598-021-89046-1

**Published:** 2021-05-04

**Authors:** Hung-Yuan Lin, Ya-Jung Chuang, Pi-Jung Lin

**Affiliations:** 1 Universal Eye Center, Zhong-Li, Taiwan, Republic of China; 2 Ophthalmology Department, Shanghai Ruidong Hospital, Shanghai, China; 3 Universal Eye Center, Long-Tan, Taiwan, Republic of China; 4 Universal Eye Center, Taipei, Taiwan, Republic of China

**Keywords:** Diseases, Optics and photonics

## Abstract

Currently, there is no clear consensus in cataract surgery if low compared to high energy femto-lasers may enable better capsulotomy quality and induce lower inflammatory response. Therefore, the aim of this study was to compare the intra-operative outcomes achieved with high and low pulse energy femtosecond laser systems for cataract surgery. The charts of 200 eyes of 200 patients aged 68.3 ± 10.3 years who had undergone femtosecond laser-assisted cataract surgery using either group 1 high pulse energy: LenSx (Alcon Laboratories) (N = 100) or group 2 low pulse energy: FEMTO LDV Z8 (Ziemer) (N = 100) laser were reviewed retrospectively. Integrity of anterior capsulotomy, classified as (1) complete (free-floating or with minor microadhesions), (2) incomplete and (3) with capsular tears, intraoperative completeness of the clear corneal incisions (CCI, main incision and side port), incidences of intraoperative miosis and incidence of subconjunctival hemorrhage were evaluated and compared between the two groups. The proportion of complete capsulotomies was significantly higher in the group 2 than the group 1 (100% vs 94%; p = 0.03). The incidences of intraoperative miosis (0% vs 19%) and subconjunctival hemorrhage (1% vs 63%) were significantly lower in the group 2 than the group 1 (p < 0.001). Completeness of the main incision was comparable (97% vs 95%; p = 0.721) between the two groups. Although not statistically significant, the completeness of side-port incision was slightly better in the group 2 than the group 1 (91% vs 86%). Low energy laser system performed significantly better in terms of completeness of capsulotomy, intraoperative miosis and sub-conjunctival hemorrhage, compared with high energy laser; the CCI outcomes were comparable.

## Introduction

One of the main challenges in phacoemulsification surgery is achieving a continuous curvilinear capsulorhexis^1,2^. Femtosecond laser-assisted cataract surgery (FLACS) has been advocated due to its ability to create a more precise and circular capsulotomy, greater predictability in achieving the desired size and centration of capsulotomy and in turn effective lens position (ELP) as compared to the manual procedure^3,4^. Despite the highly precise capsulotomy outcomes, the occurrence of capsular tags, capsule rupture, associated with FLACS created capsulotomy is not uncommon^4–6^. In addition, femtosecond laser pre-treatment has been reported to be associated with significant intra-operative pupil miosis (pupil diameter ≤ 6 mm) in up to 32% eyes^7–9^.

Femtosecond lasers employ two different patterns of photodisruption: high-energy pulses (µJ) coupled with low-frequency (kHz) and low-energy pulses (nJ) coupled with high-frequency (MHz). With a high pulse energy, greater spacing between the spots is employed, whereas the low pulse energy laser allows greater number of overlapping smaller sized spots^10^. Unlike most femtosecond laser systems, the FEMTO LDV Z8 femtosecond laser employs low pulse energy (nJ) and a high repetition rate in the MHz range. Previous studies have reported greater incidence of capsular irregularities and collateral damage using high pulse energies^11^. It has been hypothesized that low energy femtosecond lasers may enable smoother capsulotomy and induce minimal release of inflammatory mediators. Furthermore, femtosecond lasers employ different patient interfaces for the docking step. The use of curved contact interfaces has been found to increase IOP because of corneal compression during the applanation process and create corneal folds during laser cataract surgery leading to incomplete capsulotomy. In contrast, fluid-filled liquid interfaces have been found to prevent corneal folds, improve globe stability, reduce subconjunctival hemorrhage, and lower IOP rise^12,13^. Therefore, this study was aimed at comparing the intra-operative outcomes achieved with Ziemer Z8 (a low energy laser equipped with liquid patient interface) and LenSx (a high energy laser equipped with curved contact patient interface) for cataract surgery.

## Materials and methods

This single-center retrospective comparative analysis included 200 eyes of 200 cataract patients that had undergone FLACS between 2015 to 2018 at our facility. Surgeries before January 2017 were performed using LenSx (Alcon Laboratories, Inc., Fort Worth, TX) laser and surgeries after this date were performed using FEMTO LDV Z8 (Ziemer Ophthalmic Systems AG, Port, Switzerland) laser platform. Moving backward from this date, consecutive case records of 100 eligible patients were included in the LenSx laser group. For the Ziemer Z8 group, we moved forward from the date Ziemer Z8 surgeries were initiated at our facility and collected consecutive case records of 100 eligible patients that meet the recruitment criteria for the laser platform. One eye per patient was included in the study; if both eyes of a patient met the recruitment criteria, the case record of the first eye that was recruited into the study was analyzed.

All surgeries were performed by an experienced surgeon (HYL) at Universal Eye Center, Zhong-Li, Taiwan. The study was conducted in accordance with the tenets of the Declaration of Helsinki for human research and was approved by the Institutional Review Board of Antai Tian-Sheng Memorial Hospital, Taiwan (18-024-B) with a waiver of consent because the data were collected as a part of normal practice care provision.

### Inclusion and exclusion criteria

Patients older than 18 years with cataract were eligible to be included in the study. Exclusion criteria included history of glaucoma, retinal detachment, corneal pathology, significant irregular astigmatism, abnormal iris, macular degeneration or retinopathy, lens/zonular instability, previous ocular surgery or trauma, poorly dilating pupil in the operative eye and patients with diabetes mellitus (DM) with uncontrolled fasting blood sugar level (> 200 mg/dl). Patients with complicated or hard cataracts (white or black), which in the experience of investigators could influence study outcomes were also excluded. To account for the initial learning curve, first 50 eyes were excluded for the respective lasers.

### Femtosecond laser platforms

The LenSx femtosecond laser platform uses a wavelength of 1030 nm, pulse duration of 600–800 femtoseconds, maximum pulse energy of 15 μJ and pulse repetition rate of 50 kHz. It employs three-dimensional spectral domain optical coherence tomography (OCT) and video microscope to perform image guided FLACS. This laser system uses curved lens applanating patient interface with vacuum docking^14,15^.

The FEMTO LDV Z8 femtosecond laser system utilizes the concept of overlapping low-energy near-infrared (1030 nm) femtosecond laser pulses delivering energy in the nano-joule range (< 1 µJ). With a pulse duration of 250 femtoseconds, the laser runs in the MHz range applying up to 1 billion pulses per surgery. The laser system’s integrated three-dimensional high-resolution spectral-domain OCT imaging system enables visualization/identification of the precise location of ocular structures intraoperatively. It uses a non-applanating, fluid filled patient interface with vacuum docking^16,17^.

To achieve optimal outcomes, default settings recommended by respective manufacturers were used for both the laser systems. Table 1 enlists the default settings used for both the femtosecond lasers.

**Table 1:** Settings of the laser systems FEMTO LDV Z8 and LenSx.

Surgery step	Parameter	FEMTO LDV Z8	LenSx
Capsulotomy	Diameter	5.7 mm	5.3 mm
	Laser power	110%	6 µJ
	Resection height	0.6 mm	0.5 mm
Lens fragmentation	Diameter	5.8 mm	4.0 mm
	Laser power	100%	
	Number of segments	4	4
Main incisions	Laser power	115%	6 µJ
	Width	2.4 mm	2.4 mm
Paracenthesis	Laser power	115%	5 µJ
	Width	1 mm	0.9 mm

### Surgical procedure

All procedures were performed under topical anesthesia. Pupil dilation during the surgery was performed using a combination of phenylephrine and tropicamide eye drops (Mydrin-P Eye Drop, Santen.) instilled into the eye 30, 25, 20,15, and 10 min prior to surgery. Ketorolac 0.5% ophthalmic solution (Acular LS; Allergan, Inc., Irvine, CA) was instilled 4 times/day 3 days before surgery. Femtosecond laser-assisted pre-treatment included lens fragmentation and capsulotomy, clear corneal incision (CCI), and side incisions in both the groups using the femtosecond laser settings enlisted in Table 1, followed by phacoemulsification of the lens nucleus and aspiration of lens cortex. Finally, a foldable intraocular lens (IOL) was implanted into the capsular bag. All surgical procedures were video recorded as a routine procedure. All eyes received the same standardized post-operative regimen for both the laser groups. In addition, laser-assisted astigmatic keratotomy was performed in patients with significant amounts (between -0.75D and -2D) of pre-existing corneal astigmatism [43% (43/100 eyes) in the LenSx laser group and 56% (56/100 eyes) in the Z8 laser group, however, there was no statistically significant difference between the two groups, X^2^ (1, N = 200) = 2.89, p = 0.09].

For LenSx laser group, the femtosecond laser portion was performed in a different room adjacent to the operating room. In contrast, the Z8 laser platform allowed the whole surgical procedure (both laser and manual parts of the surgery) to be performed in the same operating room and under sterile conditions.

### Study outcome measures

The videos of all surgeries were retrospectively reviewed, and the following data was collected. Integrity of anterior capsulotomy was recorded as: (1) complete treatment pattern (complete 360° cut as free-floating capsulotomy or with minor microadhesions, not influencing the free-float), (2) incomplete treatment pattern (femtosecond laser cut not visible in a certain section of the anterior capsulotomy) and (3) with capsular tears. CCI parameters included completeness of main incision and side ports, evaluated based on resistance experienced by the surgeon when entering the incision. If the incision was easily opened by blunt tip of the viscoelastic cannula (Viscoat, (Alcon Surgical, Inc., Fort Worth, TX), it was defined as ‘complete’ and if the resistance was such that the surgeon had to use a knife to open the incision, it was considered as ‘incomplete’. Additionally, the surgeon also recorded the presence of sub-conjunctival hemorrhage and intraoperative miosis (defined as the inability to visualize the capsulotomy margin before opening the main incision). Cases where the capsulotomy was not visible anymore after the laser pre-treatment, were considered as cases manifesting miosis.

### Statistical analysis

Data analysis was performed using SPSS software for Windows version 17.0 (SPSS Inc., Chicago, IL). Categorical data were analyzed using Chi-Square test; however, Fisher’s exact test was used in cases where more than 20% of the cells had expected cell values of less than 5 or if the expected value in any cell of the contingency table was less than 1. In case of continuous variables, normality of the data was checked using Kolmogorov–Smirnov test and quantile–quantile plots. For normally distributed scale data, independent-sample t test was used to compare the means between the two groups. All P-values were two-sided and were considered statistically significant when less than 0.05. The sample size was calculated using literature reported incidence of free-floating capsulotomies achieved with LenSx laser (average 88.4%)^2,18–20^ and Ziemer Z8 laser (average 98.7%)^21,22^. Using these reported values for the two lasers, sample size calculations were performed with two-sided alpha of 0.05 and 80% power which revealed a total sample size of 190 (95 in each group). Accordingly, we chose a sample size of 100 in each group.

## Results

A total of 200 eyes of 200 patients (94 males and 106 females) with a mean age of 68.3 ± 10.3 years were included in the study. The mean age of the participants in the LenSx and Z8 group were 64.4 ± 9.9 and 61.7 ± 11 years respectively. The difference between the groups in terms of age was not significant (t(196) = 1.79; p = 0.07).

**Figure 1:**
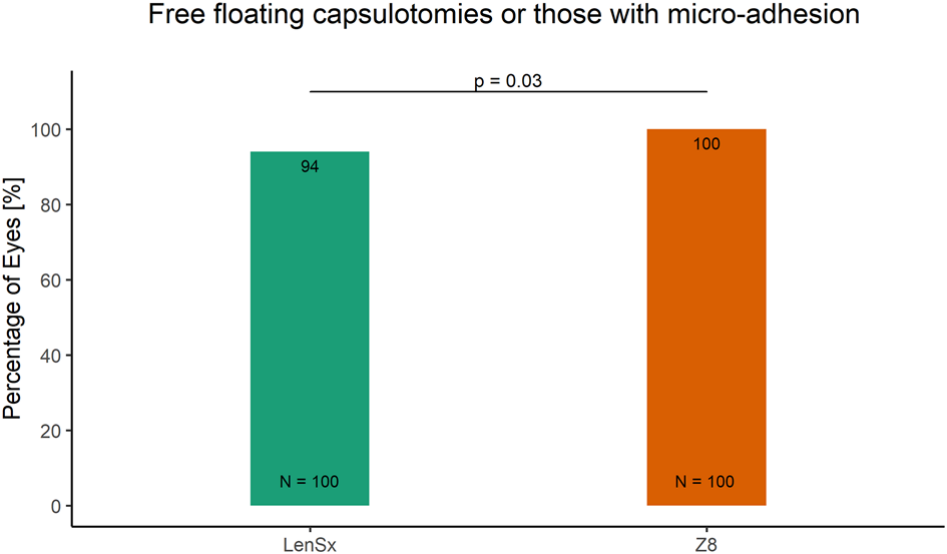
The proportion of eyes with free-floating capsulotomies or those with micro adhesions in the LenSx and the Z8 laser group.

The number of free-floating capsulotomies was significantly higher (100%) in Z8 group as compared to 94% in the LenSx group (p = 0.03, two-sided Fisher’s exact test Fig. 1). Other capsulotomy parameters including incomplete treatment pattern (0% vs 5% in Z8 and LenSx respectively) and capsular tears (0% vs 1% in Z8 and LenSx respectively) were also found to be lower in Z8 laser group; however, the differences were not statistically significant (p > 0.05, two-sided Fisher’s exact test).

**Figure 2:**
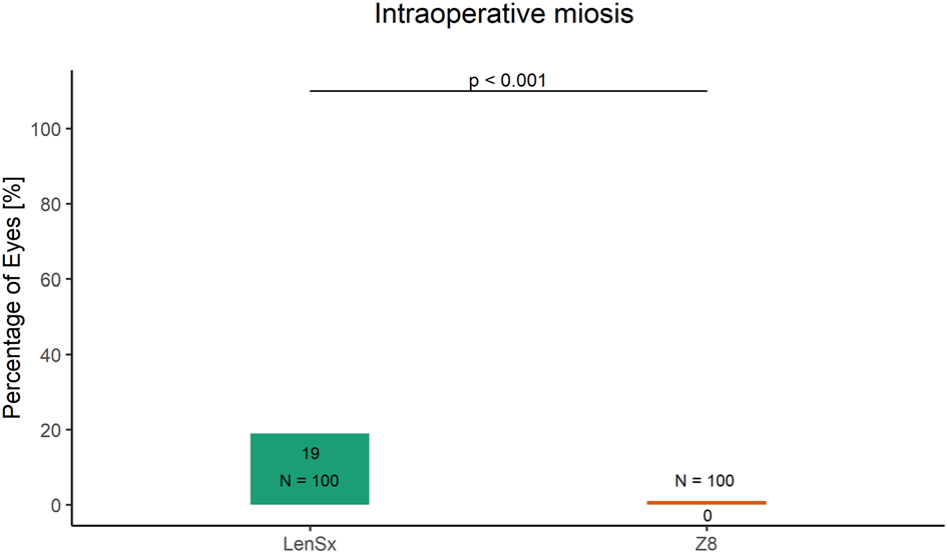
The proportion of eyes with intraoperative miosis in the LenSx and the Z8 laser group.

**Figure 3:**
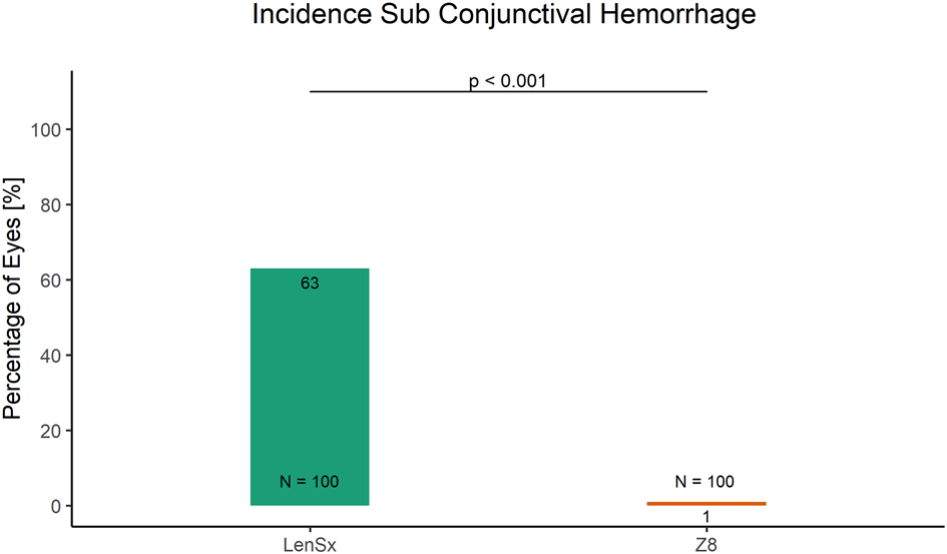
The proportion of eyes with incidence of subconjunctival hemorrhage in the LenSx and the Z8 laser group.

Compared to 19% eyes treated with LenSx laser system, none of the eyes experienced intra-operative miosis in the Z8 laser group (*X*^2^ (1, *N* = 200) = 18.843, p < 0.001, Fig. 2). The LenSx laser group had a significantly greater incidence of subconjunctival hemorrhage, compared to Z8 group (63% vs 1%: *X*^2^ (1, *N* = 200) = 85.501, p < 0.001, Fig. 3).

**Figure 4:**
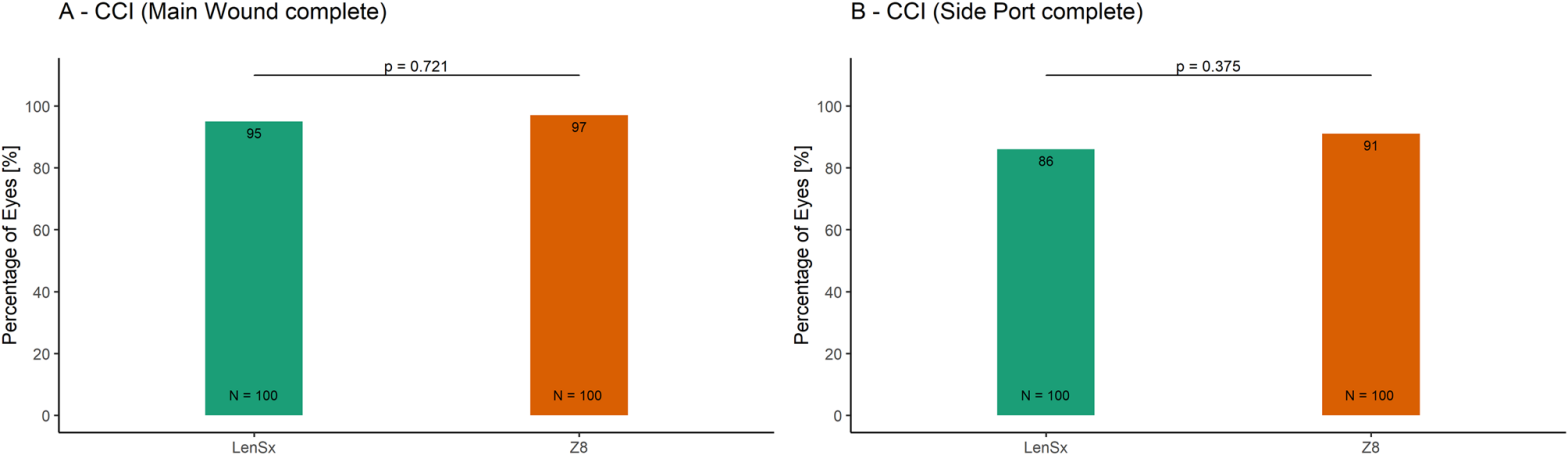
The proportion of eyes with complete CCI in the LenSx and the Z8 laser group (**A**) Main incision. (**B**) Side-port.

Completeness of the main incision was observed to be comparable between the two groups (97% in the Z8 group and 95% in LenSx group; p = 0.721). The outcomes for the completeness of side-port incision were slightly better in the Z8 group (91%) than in the LenSx group (86%), however the difference was not statistically significant (*X*^2^ (1, *N* = 200) = 0.786, p = 0.375, Fig. 4).

## Discussion

While numerous studies have provided substantial evidence for the safety and efficacy of FLACS, several complications specific to femtosecond lasers have been documented^9^. Unlike high pulse energy and low frequency femtosecond lasers which emit energy in the microjoule range, the low energy laser combines low pulse energy (in nano Joules) with a high frequency rate to achieve overlapping precisely placed laser spots which has been hypothesized to allow smooth incisions^23^, capsulotomy^4^ and minimal inflammatory response^24^.

The current study was designed to evaluate if there are any differences in high and low pulse energy femtosecond lasers in terms of integrity of capsulotomy, completeness of CCI, incidence of sub-conjunctival hemorrhage and intraoperative miosis.

### Capsulotomies

Femtosecond laser enables consistently shaped and well-centered capsulotomies which helps maintain accurate IOL positioning within the capsular bag, thus improving postoperative refractive outcomes^3^. However, several capsular irregularities may still occur with laser-assisted capsulotomies including, capsular microadhesions, incomplete and non-continuous treatment pattern, which may compromise the surgical benefits of femtosecond laser technology^17^. Variations in intrinsic laser settings, most importantly pulse energy, have been shown to affect the morphology and capsular irregularities associated with femtosecond laser-assisted capsulotomy cutting edges^11^. A comparison of capsulotomy outcomes with high (15 µJ) and lower pulse energy (7 µJ) revealed more capsular irregularities (for e.g., anterior tags, adherence of the capsule to the underlying lens material, prominent demarcation line) with higher pulse energy settings using LenSx Laser System, (Alcon Laboratories, Inc.) or Lensar Laser System, (Lensar, Inc.)^11,25^. In contrast, cutting edges of capsulotomies performed using lower pulse energy (5 µJ) were found to be smoother and rounder^11^. The curved contact patient interfaces can create corneal folds that have been associated with incomplete capsulotomies during laser cataract surgery^13^.

Williams G.P. et al. showed that the low energy FEMTO LDV Z8 created circular, rupture resistant and smooth capsulotomies in both porcine and human globes^4^. Pisciotta A. et al. reported highly continuous morphology of capsule edges which was attributed to the low energy/high frequency properties of the laser pulse, combined with an overlapped pulse pattern^26^. Furthermore, Schwarzenbacher et al. have demonstrated that especially low pulse energy lasers do not significantly increase the level of inflammatory cytokines (interleukin), which indicates low damage to the surrounding tissue^24^. Another article by Verdina T. et al. reported, that low energy capsulotomy group had higher circularity and stability of capsulotomy diameter compared with the standard bimanual microincision cataract surgery technique over the 18-month observation period and was also associated with lower PCO rates^27^. Also, a report evaluating learning curve with the FLACS found three capsule related complications with the low energy femtosecond laser during initial 60 cases, and none after overcoming the learning curve^22^.

As in line with literature, in this study the lower pulse energy settings and the high frequency of Z8 FLACS system and thus the overlapping precisely placed laser spots compared with LenSx showed significantly better capsulotomy outcomes. The same effect was also seen in the lower number capsular tears and incomplete treatment pattern.

### Completeness of CCI

In the present study, we also investigated the effect of two laser systems on the completeness of main and side port incisions. The results indicated slightly higher percentage of eyes with complete CCIs in Z8 group (although the difference was not statistically significant), compared to LenSx group. It is important to note that the completeness and ease of opening of corneal incisions depend on their proximity to the limbus. While the limbal incisions are desirable with regard to the surgically induced astigmatism, they might be difficult to open as the cornea gets thicker and less transparent towards the periphery. The incisions created by FEMTO LDV Z8 are mostly placed closer to limbus as compared with the LenSx incisions, which are created rather corneal, due to different centration and docking mechanisms. Therefore, the comparable outcomes between two laser systems in terms of completeness of CCI and side-ports must be interpreted considering, that the distance to limbus was not taken into account when the incisions quality was evaluated.

### Incidence of sub-conjunctival hemorrhage

Subconjunctival hemorrhage after femtosecond laser surgery has been associated with the docking process (scleral contact of the interface) and strong suction pressure applied at the patient interface^9^. In an experimental study, liquid optical interface (LOI) performed better in terms of elimination of corneal folds, improved globe stability, reduced subconjunctival hemorrhage and lower IOP elevation, compared to the curved contact lens (CCL) interface system^13^. Consistent with these findings, the incidence of subconjunctival hemorrhage was found to be significantly higher with contact corneal applanation based LenSx group, compared to Z8 group that employs non-applanation fluid filled patient interface (63% vs 1%; p < 0.001) in this study. Similarly, previous publications also reported significantly lower incidence of sub-conjunctival hemorrhage with liquid interface systems, compared to CCL interface system^15,28^. With the availability of advanced presbyopia correcting intraocular lens (IOLs) and changing practice patterns, there is a gradual shift in age of the patients undergoing cataract/refractive lens exchange surgeries. The younger and cosmetically more aware patients are likely to value the lower probability of post-FLACS subconjunctival hemorrhage with low energy Z8 laser, thereby increasing patient satisfaction in such patient population.

### Intraoperative miosis

Intraoperative pupil miosis is the most frequently reported complication during FLACS. It is hypothesized that pupil miosis is linked to the release of prostaglandin and other inflammatory mediators by the ciliary body in response to dissipation of laser energy, leading to increase in temperature, shock waves and vibrations^29^. Laser pulse energy is thought to play an important role in the degree of inflammatory response generated by the femtosecond laser^30–33^. It has been reported that decrease in applied laser pulse energy reduces the damage to surrounding tissues^11,34^. Supporting this theory, previous studies have reported a substantially high incidence of intraoperative miosis (as high as 32%) with the use of high energy femtosecond laser systems^7–9^. Consistent with the literature, the incidence of intraoperative miosis was found to be significantly higher in LenSx treated eyes, compared to Z8 group (19% vs 1%; p < 0.001) in this study. Apparently, the low energy Z8 laser would induce minimal collateral damage to surrounding tissue, thus ensuing lower amounts of prostaglandin release, thereby reducing the risk of pupillary miosis during the surgery^32^, compared with high energy laser systems^31,33,35,36^. In agreement with these findings, a recent study reported no significant changes in pre- and post-laser treated pupil area with low energy Z8 FLACS system^37^. Conversely, Diakonis et al. revealed significant pupillary miosis with 3 different high energy femtosecond laser platforms^38^.

Although, we did not evaluate the levels of prostaglandins released following treatment with the two laser systems in this study, a meeting abstract presented at ARVO 2018 annual meeting reported only a slight increase in prostaglandins with a low energy femtosecond laser system (median value: 20.15 pg/ml after 5 min of laser pre-treatment)^24^. Whereas, another publication reported markedly high levels of prostaglandins with the use of high energy laser systems (mean values: 330.6 pg/ml after full treatment including capsulotomy and fragmentation; 362.4 pg/ml after laser capsulotomy)^38^.

Another likely reason for significantly high miotic effect observed with the LenSx laser group could be due to the extended time lapse between femtosecond laser pretreatment and the further surgical steps involved with this laser system. Conventional non-mobile laser systems are usually placed in a different room and after the pre-treatment the patient needs to be transferred to the sterile operating room where phacoemulsification is performed, thus necessitating a transition time for patient transfer and sterile draping. In some settings, the patient may have to move up and the operating room may be a hallway away from the laser room. Whereas, with mobile Z8 laser system (housed in the same room), phacoemulsification can be performed immediately following laser application, thereby causing no delay between femtosecond laser pre-treatment and phacoemulsification^39^. In addition, the entire procedure is carried under sterile conditions of the operating room. As such, housing the femtosecond laser and the operating table in the same room is most suitable^40^, and must become the only accepted option.

Pre-treatment with topical NSAIDs has been suggested to lower prostaglandin release, thus may have potential benefit of preventing pupil miosis^32,36,41^. In this study, preoperative NSAID eyedrops regimen was uniform for the two laser groups, thus eliminating any potential bias due to differences in NSAID regimen responsible for significant difference in intraoperative miosis between the two laser groups. In both groups all eyes received NSAIDs drops combined with the mydriatics prior to surgery. Given the lower incidence of intraoperative miosis in the Z8 group, patients with documented NSAID allergy may benefit from the use of the low energy Z8 laser. The hypothesis is supported by the findings of Mirshahi et al., that did not detect statistically significant changes in the pupil area after laser pre-treatment with the low energy FEMTO LDV Z8^37^.

### Limitations

Although patient demographics such as age and sex were comparable between the two laser groups; we did not compare preoperative ocular characteristics such as axial length, nuclear sclerosis grade, co-pathologies and visual outcome which may have influenced the study outcomes and therefore could be a potential limitation of this study. Also, the two lasers were operated using the default settings recommended by the manufacturers in an attempt to achieve optimal outcomes with the respective lasers (Table 1). Besides the resection height of the capsulotomy being centered on the anterior capsule, completeness of the capsulotomy is more likely a function of overlapping adjacent laser spots^23^. The variation in the anterior capsule thickness of a few microns is unlikely to affect the completeness of the capsulotomy, although it cannot be ruled out completely^42^. Future studies with comparable capsulotomy diameters may help validate the findings of the present study. The two lasers place incisions at different distances to the limbus. Incisions created by LenSx are rather corneal, whereas the incisions created by FEMTO LDV Z8 are mostly placed closer to the limbus. The corneal incisions are expected to be easier to open, and incisions closer to the limbus are expected to be harder due to less transparent cornea in the periphery. Thus, the comparable outcomes between two laser systems in terms of completeness of CCI and side-ports must be interpreted considering that default laser settings were used and the distance to limbus was not taken into account when the incisions quality was evaluated. Another limitation of this study could be that the visualisation of the capsulotomy margin was chosen as criterion of absence of intraoperative miosis, instead of directly measuring the pupil area or diameter.

## Conclusion

To summarize, low energy Z8 laser system performed significantly better in terms of completeness of capsulotomy, intraoperative miosis and sub-conjunctival hemorrhage, compared with high energy LenSx laser under default laser settings recommended by the manufacturers; however, CCI outcomes were found to be comparable. The authors associate these findings to the low energy laser being gentler on the ocular tissue and causing low inflammation. Future technological innovations and prospective studies in different aspects of low energy femtosecond lasers are expected to further improve the safety and effectiveness of the procedure and allow faster visual rehabilitation and greater patient satisfaction.
